# Prognostic Value of the Noble and Underwood Score in Patients with Non-Small Cell Lung Cancer Undergoing Surgical Resection

**DOI:** 10.7150/jca.101320

**Published:** 2024-10-14

**Authors:** Soomin An, Wankyu Eo, Dae Hyun Kim, Sookyung Lee

**Affiliations:** 1 Department of Nursing, Dongyang University, Gyeongbuk, Republic of Korea.; 2 College of Medicine, Kyung Hee University, Seoul, Republic of Korea.; 3 Department of Thoracic Surgery, Kyung Hee University Hospital at Gangdong, Seoul, Republic of Korea.; 4 Department of Clinical Oncology, College of Korean Medicine, Kyung Hee University, Seoul, Republic of Korea.

**Keywords:** carcinoma, non-small cell lung, Inflammation, Nutritional index, Pulmonary surgical procedures

## Abstract

**Background:** This retrospective study aimed to evaluate the clinical utility of the Noble and Underwood (NUn) score as a prognostic marker for overall survival (OS) in patients with stage I to IIIA non-small cell lung cancer (NSCLC). The NUn score is a novel composite marker that integrates C-reactive protein (CRP), serum albumin (ALB) levels, and white blood cell (WBC) count to provide a comprehensive assessment of systemic inflammation and nutritional status.

**Methods:** We included patients with stage I to IIIA NSCLC and assessed the NUn score, calculated using CRP, ALB levels, and WBC count. Hazard ratios for OS were determined using Cox regression analysis. The predictive performance of the models was evaluated through metrics such as area under the curve (AUC), concordance index (C-index), integrated AUC (iAUC), integrated discrimination improvement (IDI), continuous net reclassification index (cNRI), and decision curve analysis (DCA).

**Results:** The median age of the patients was 69 years, and 63.1% of patients were men. The cohort included 152 (63.1%) patients with stage I disease, 54 (22.4%) with stage II disease, and 35 (14.5%) with stage IIIA disease. In the multivariate Cox regression analysis, the NUn score, age, American Society of Anesthesiologists Physical Status, tumor-node-metastasis (TNM) stage, and pleural invasion emerged as independent prognostic factors for OS, forming the NUn model. The C-index and iAUC of the NUn model (0.832 and 0.802, respectively) outperformed those of the baseline model based solely on TNM stage. The NUn model also demonstrated superior discriminative capacity compared with the baseline model using metrics such as AUC, IDI, cNRI, and DCA at 3 and 5 years after surgery. Calibration of the nomogram based on the NUn model showed good accuracy.

**Conclusions:** These findings underscore the prognostic significance of the NUn score in predicting OS among patients with stage I to IIIA NSCLC by integrating markers of inflammation and nutritional status. The NUn model, which integrates the NUn score with other clinical variables, exhibited superior discriminative ability compared with TNM stage alone. These findings highlight the potential of the NUn score as a valuable tool in personalized care for patients with NSCLC. Further external validation with independent cohorts is necessary to confirm the model's applicability to other populations.

## Introduction

Surgery remains the best option for patients with tumor-node-metastasis (TNM) stage I to IIIA non-small cell lung cancer (NSCLC) 1. Despite substantial advances in surgical techniques and adjuvant therapy, the prognosis for these patients remains far from satisfactory 2. Therefore, identifying key prognostic factors that can identify high-risk patients is crucial for improving clinical outcomes.

Demographic parameters, such as age, sex, performance status, and smoking history, are significant determinants of survival of patients with NSCLC 3-6. The TNM staging system remains the primary predictor of survival in these patients 4, 6-8. Additionally, clinicopathological factors such as histology, tumor size, pleural invasion, vascular invasion, lymphatic invasion, type of surgery, and residual disease have been recognized as important predictors of survival of patients with NSCLC 2, 4-11.

C-reactive protein (CRP) is an acute-phase reactant that is highly sensitive to systemic inflammatory responses and is one of the most commonly used inflammatory markers in clinical settings 11-18. CRP has been identified as a significant determinant of survival outcomes of patients with NSCLC 3, 11, 16-18. Similarly, serum albumin (ALB) is another inflammation-related nutritional biomarker and is recognized as a potent prognostic factor 1, 19-21. ALB has also been suggested as a significant determinant of oncological outcomes in patients with NSCLC 3, 22-25.

Both serum CRP and ALB are included in several prognostic formulas, such as the CRP-ALB ratio (CAR) 26, Osaka prognostic score (OPS) 27, C-reactive protein-albumin-lymphocyte (CALLY) index 3, modified Glasgow prognostic score (mGPS) 28, and Noble and Underwood (NUn) score 29, In NSCLC, CAR, CALLY index, and mGPS have been reported as predictors for overall survival (OS) 3, 26, 28.

The NUn score, introduced by Noble and Underwood, is a logistic regression model based on CRP and ALB levels and white blood cell (WBC) counts. These three parameters are associated with the systemic inflammatory response. Originally measured on postoperative day 4, the NUn score has been reported to be a predictor of anastomotic leakage and major complications following surgery 29. The NUn score has subsequently been validated as a predictor of anastomotic leakage in patients with esophageal cancer, although a consensus on its predictive value has not yet been reached 30-32.

Recently, the NUn score has been validated as a simple measure for predicting long-term survival outcomes after surgery for gastric cancer 33-35. However, the clinical value of the NUn score in predicting long-term survival outcomes in tumors other than gastric cancer has not been reported. Therefore, in the present study, we aimed to determine the accuracy of the NUn score as a predictor of OS in patients with stage I to IIIA NSCLC who underwent curative-intent surgical resection.

## Methods

### Patients

Electronic medical records of consecutive patients with NSCLC who underwent surgical resection between January 2010 and March 2020 at Kyung Hee University Hospital in Gangdong were reviewed. Chest and abdominopelvic computed tomography and positron emission tomography-computed tomography are regular components of standard cancer staging.

The inclusion criteria were as follows: (i) primary NSCLC 36, (ii) stage I to IIIA according to the 8th edition of the lung cancer stage classification 37, and (iii) microscopic margin-negative resection 38. The exclusion criteria were as follows: (i) anti-cancer treatment for NSCLC before surgery, (ii) stage IIIB or IV disease, (iii) concurrent second malignancies or previous malignancies within the last 5 years, and (iv) active infections or connective tissue diseases undergoing treatment.

This study was approved by the Institutional Review Board (IRB) of Kyung Hee University Hospital in Gangdong (2024-07-005). Given the retrospective nature of this study, the requirement for informed consent was waived by the IRB.

### Clinical characteristics

The clinicopathological variables collected and analyzed in this study were age, sex, smoking history, height, body weight, body mass index (BMI), American Society of Anesthesiologists Physical Status (ASA-PS), type of surgery, histology, tumor size, extent of primary tumor, lymph node invasion, TNM stage, pleural invasion, lymphatic invasion, vascular invasion, perineural invasion, and microscopic residual disease status. Pleural invasions were categorized from 0 to 3 39. The laboratory studies analyzed in this study included blood chemistry (ALB and CRP) and hemograms (WBC count, absolute neutrophil count [ANC], absolute monocyte count [AMC], absolute lymphocyte count [ALC], hemoglobin level, platelet count, and mean platelet volume [MPV]). Hemograms were measured using an LH 1502 impedance counter (Beckman Coulter, Inc., Miami, Florida, United States). Blood test results were obtained from tests conducted within 7 days before surgery.

All blood samples for MPV measurement were uniformly collected, handled, and processed according to local laboratory guidelines. Ethylenediaminetetraacetic acid-anticoagulated blood samples were processed at a temperature of 20°C to 25°C within 1 hour of venous sampling. Regular quality control was performed to ensure accuracy and reliability 40, 41.

The neutrophil-to-lymphocyte ratio (NLR) was calculated by dividing the ANC by the ALC. The lymphocyte-to-monocyte ratio (LMR) was calculated by dividing the ALC by the AMC. The platelet-to-lymphocyte ratio (PLR) was calculated by dividing the platelet count by the ALC. The NUn score was calculated according to the original formula by Noble and Underwood, namely: NUn score = 11.3894 + (0.005 × CRP in mg/L) + (0.186 × WBC in 10^9^/L) - (0.174 × ALB in g/L) 29. CAR was calculated by dividing the CRP by the ALB 26. The CALLY index was calculated using the formula: ALB × ALC/(CRP × 10^4^) 3. OPS was calculated by counting the number of positive findings as follows: elevated CRP (>1.0 mg/dL), low ALB (<3.5 g/dL), and low ALC (<1600/μL) 27. The mGPS was determined as follows: patients with both elevated CRP (>1.0 mg/dL) and low ALB (<3.5 g/dL) were assigned a score of 2; those with only elevated CRP (>1.0 mg/dL) were assigned a score of 1; and those without elevated CRP (≤1.0 mg/dL) were assigned a score of 0 27.

### Statistical analyses

OS was measured from surgical resection to all-cause mortality. To preserve the full spectrum of information and maintain statistical power, continuous variables were left uncategorized. This approach improves the detection of meaningful relationships between variables and minimizes the risk of overfitting, especially given our sample size. Continuous variables are less prone to overfitting and typically yield more generalizable findings. Additionally, they provide personalized and precise prognostic assessments, making them highly actionable in clinical practice 42-44. Continuous variables are presented as medians with interquartile ranges (IQRs). Correlation coefficients between NUn scores and other variables were analyzed using Spearman's rank-order correlation. Nonparametric tests, such as the Mann-Whitney U test or Kruskal-Wallis test, were employed for inter-group comparisons of the variables.

Cox regression analysis was utilized to calculate hazard ratios (HRs) for various variables. Significant variables (P < 0.05) identified in the univariate Cox regression analysis were included in the multivariate Cox regression analysis, and those that did not meet the proportional hazards assumption were excluded. Multicollinearity among variables was assessed using the variance inflation factor (VIF).

The discriminative performances of the models were evaluated using several metrics, including the concordance index (C-index) and integrated area under the curve (iAUC). Differences in the C-index between models were assessed employing 1,000 bootstrap re-samples, while differences in iAUC were tested using permutation tests with 1,000 re-samples.

The area under the curve (AUC) at both 3 and 5 years after surgery was evaluated to assess the predictive capabilities of the models. Differences between the models were analyzed using 1,000 bootstrap re-samples. Integrated discrimination improvement (IDI), continuous net reclassification improvement (cNRI), and decision curve analysis (DCA) were used to compare the predictive performance of the models for OS at 3 and 5 years after surgery. Bootstrap resampling was employed with 1,000 iterations to assess the robustness of the DCA results.

Finally, a nomogram was developed based on the established model to predict OS. A nomogram integrates multiple predictive variables into a graphical tool to estimate the probability or risk score for an outcome. Calibration curves employing 1,000 bootstrap re-samples were used for internal validation of the nomogram to ensure reliability and prevent overfitting.

All the statistical analyses were performed by a statistician among the authors. All P-values were two sided, and statistical significance was set at P-values < 0.05. Data were analyzed using the R package.

## Results

### Clinicopathological characteristics of the patients

**Figure 1:**
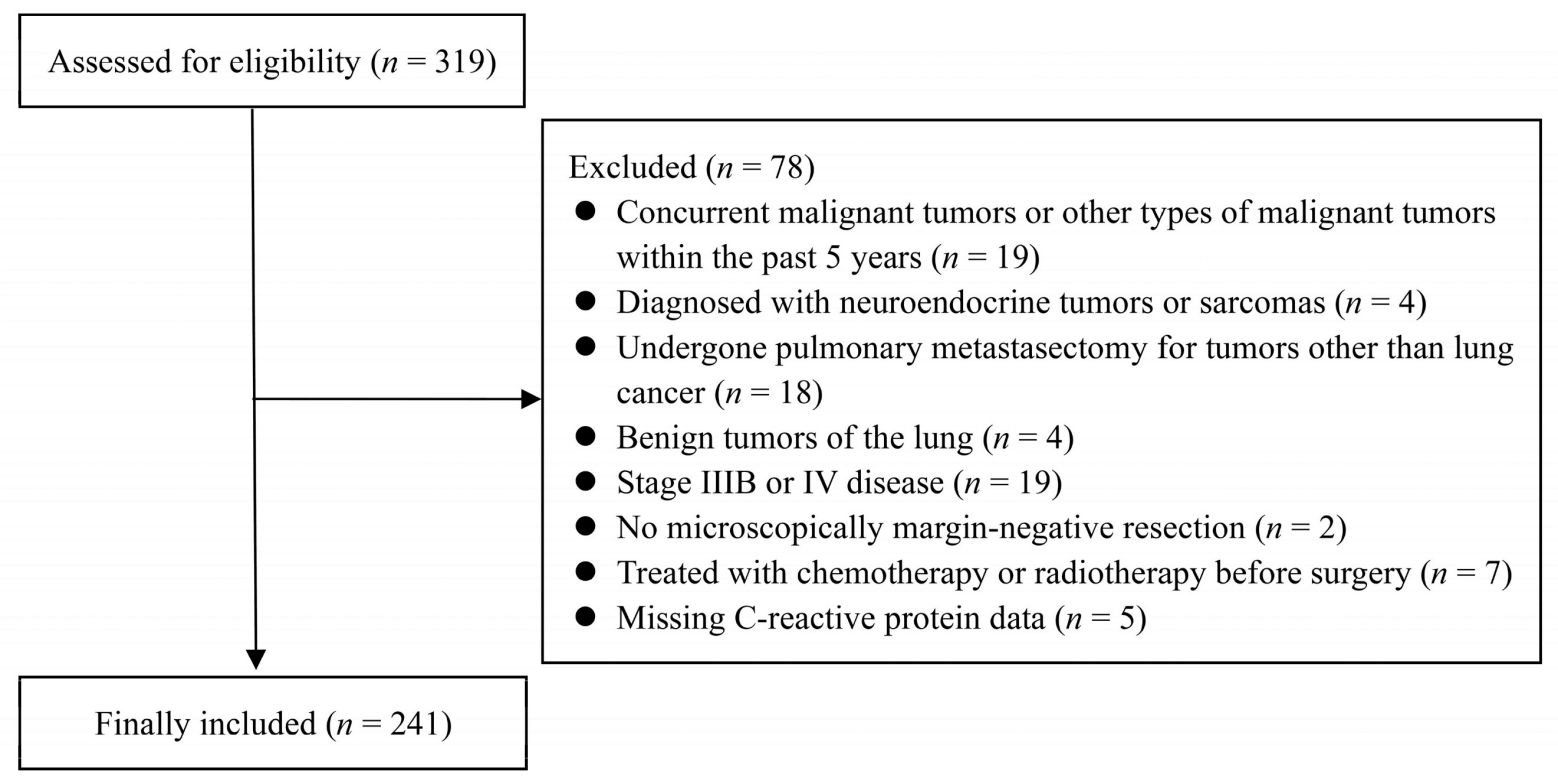
Flow Diagram: Overview of the Study Protocol.

Among 319 patients with NSCLC who underwent surgical resection, 78 were excluded, resulting in 241 patients included in the analysis (Figure 1). Most patients underwent lobectomy (76.8%, n = 185), followed by segmentectomy (21.2%, n = 51) and pneumonectomy (2.0%, n = 5). Histologically, 64 patients (26.6%) had squamous cell carcinoma, and 177 patients (73.4%) had non-squamous cell carcinoma. Regarding the disease stage, 152 patients (63.1%) had stage I; 54 (22.4%), stage II; 35 (14.5%), stage IIIA (Tables 1 and 2).

### Associations of NUn score with variables

When applying the Mann-Whitney U test or Kruskal-Wallis test, significant between-group differences in NUn scores were observed across various variables such as sex, ASA-PS, smoking history, histology, TNM stage, type of surgery, pleural invasion, and anemia (Table 1).

Strong correlations (r > 0.5) were observed between the NUn score and several variables, including ALB, CRP, WBC, ANC, AMC, and NLR (Table 2).

### Cox proportional hazard regression analysis

The median follow-up duration was 71.7 months (IQR: 55.6-94.7 months). Using univariate Cox regression analysis, age, sex, ASA-PS, smoking history, histology, tumor size, N stage, TNM stage, pleural invasion, lymphatic invasion, vascular invasion, ALB, CRP, WBC count, ANC, AMC, MPV, NLR, CAR, CALLY index, OPS, mGPS, and NUn scores were identified as significant predictors of OS (Table 3).

Several variables remained as significant determinants of OS in the multivariate Cox regression model. Age had an HR of 1.07 (P < 0.001); ASA-PS, 1.87 (P = 0.017); TNM stage, 4.00 (P < 0.001); pleural invasion, 1.34 (P = 0.031); the NUn score, 1.41 (P = 0.001) (Table 3).

The VIFs for these variables were 1.03 for age, 1.04 for ASA-PS, 1.11 for TNM stage, 1.09 for pleural invasion, and 1.04 for the NUn score, indicating low multicollinearity among the predictors. These five variables constituted the NUn model.

### Comparison between the NUn and baseline models

The discriminative power of the NUn model, which integrates the NUn score with other clinical variables, was compared with that of the baseline model based solely on the TNM stage.

The C-index for the NUn model was significantly higher than that of the baseline model (0.832 vs. 0.720, P < 0.001). Similarly, the iAUC for the NUn model was substantially higher than that for the baseline model (0.802 vs. 0.705, P < 0.001) (Table 4).

**Figure 2:**
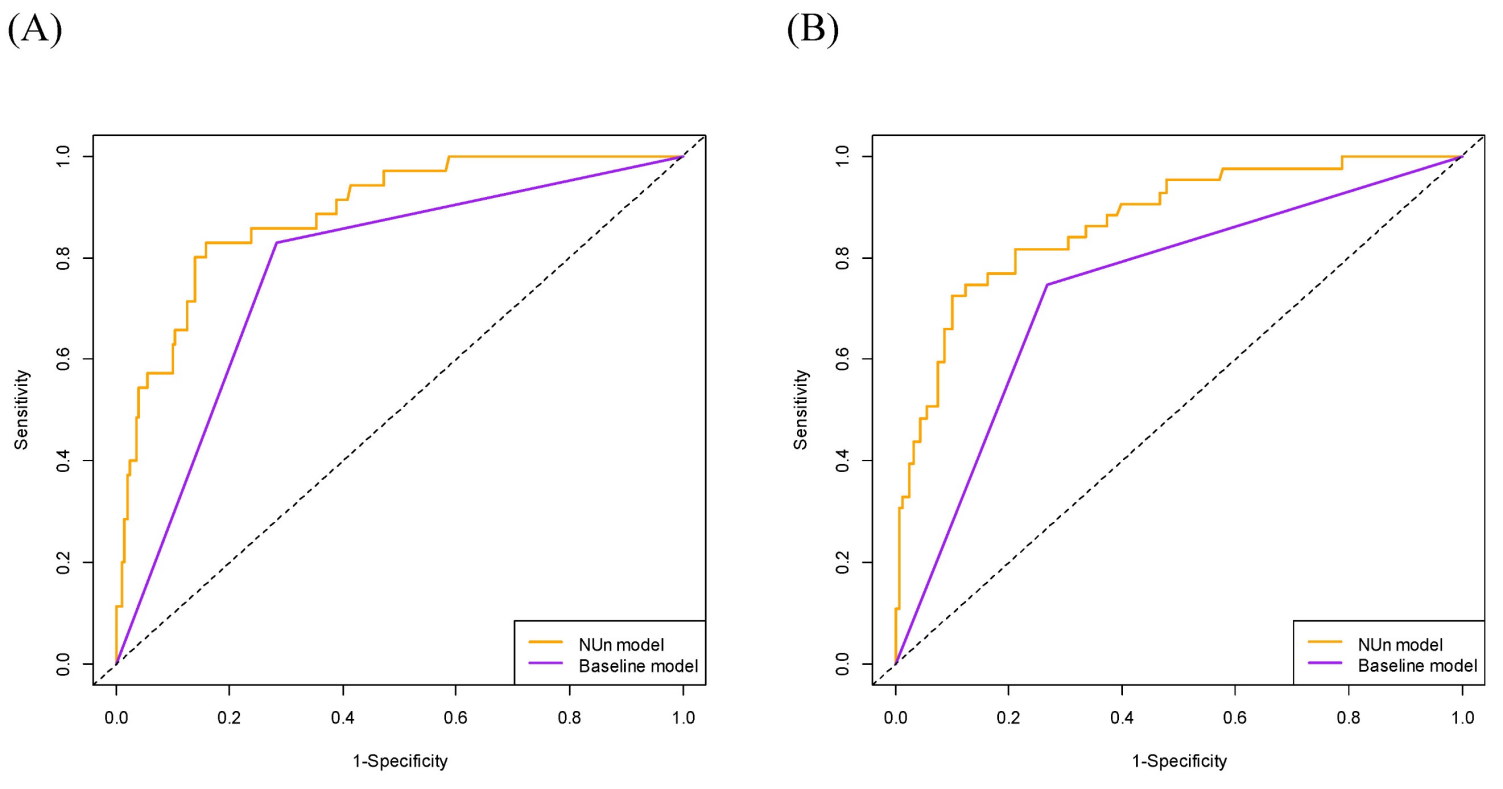
Comparison of Area Under the Curve (AUC) for Predicting 3-Year (A) and 5-Year (B) Overall Survival Between Models. The full model consisted of age, American Society of Anesthesiologists Physical Status, TNM stage, and pleural invasion and NUn scores. The baseline model relied solely on the TNM stage. TNM: Tumor-Node-Metastasis.

The 3-year OS AUC was significantly higher for the NUn model than for the baseline model (0.887 vs. 0.773, P < 0.001). Similarly, the 5-year OS AUC was significantly higher for the NUn model than for the baseline model (0.871 vs. 0.740, P < 0.001) (Table 4, Figure 2).

Using the IDI metric, there was a notable enhancement in discrimination with the NUn model compared with the baseline model at both 3 (IDI, 0.163; P < 0.001) and 5 years (IDI, 0.179; P < 0.001). Additionally, the cNRI indicated a significant improvement in reclassification with the NUn model at 3 (cNRI, 0.368; P < 0.001) and 5 years (cNRI, 0.405; P < 0.001) (Table 4).

**Figure 3:**
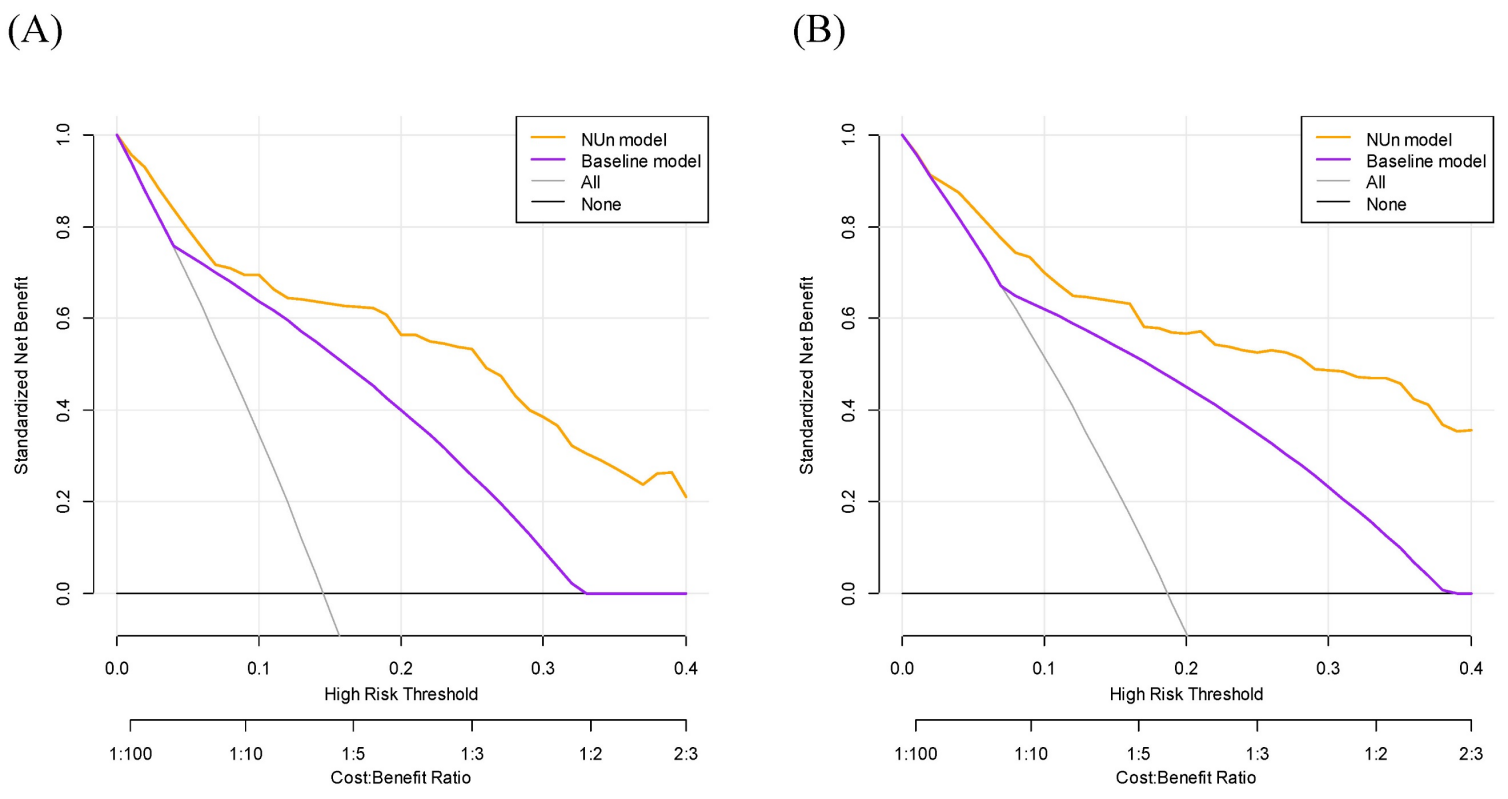
Evaluation of Survival Prediction Models: Decision Curve Analysis for 3-Year (A) and 5-Year (B) Overall Survival. The full model consisted of age, American Society of Anesthesiologists Physical Status, TNM stage, and pleural invasion and NUn scores. The baseline model relied solely on the TNM stage. TNM: Tumor-Node-Metastasis.

DCA for predicting the 3- and 5-year OS demonstrated significant differences between the two models. The NUn model provided a higher net benefit than the baseline model, suggesting a better predictive accuracy and utility in making clinical decisions (Figure 3).

### Nomogram for predicting 3- and 5-year survival

**Figure 4:**
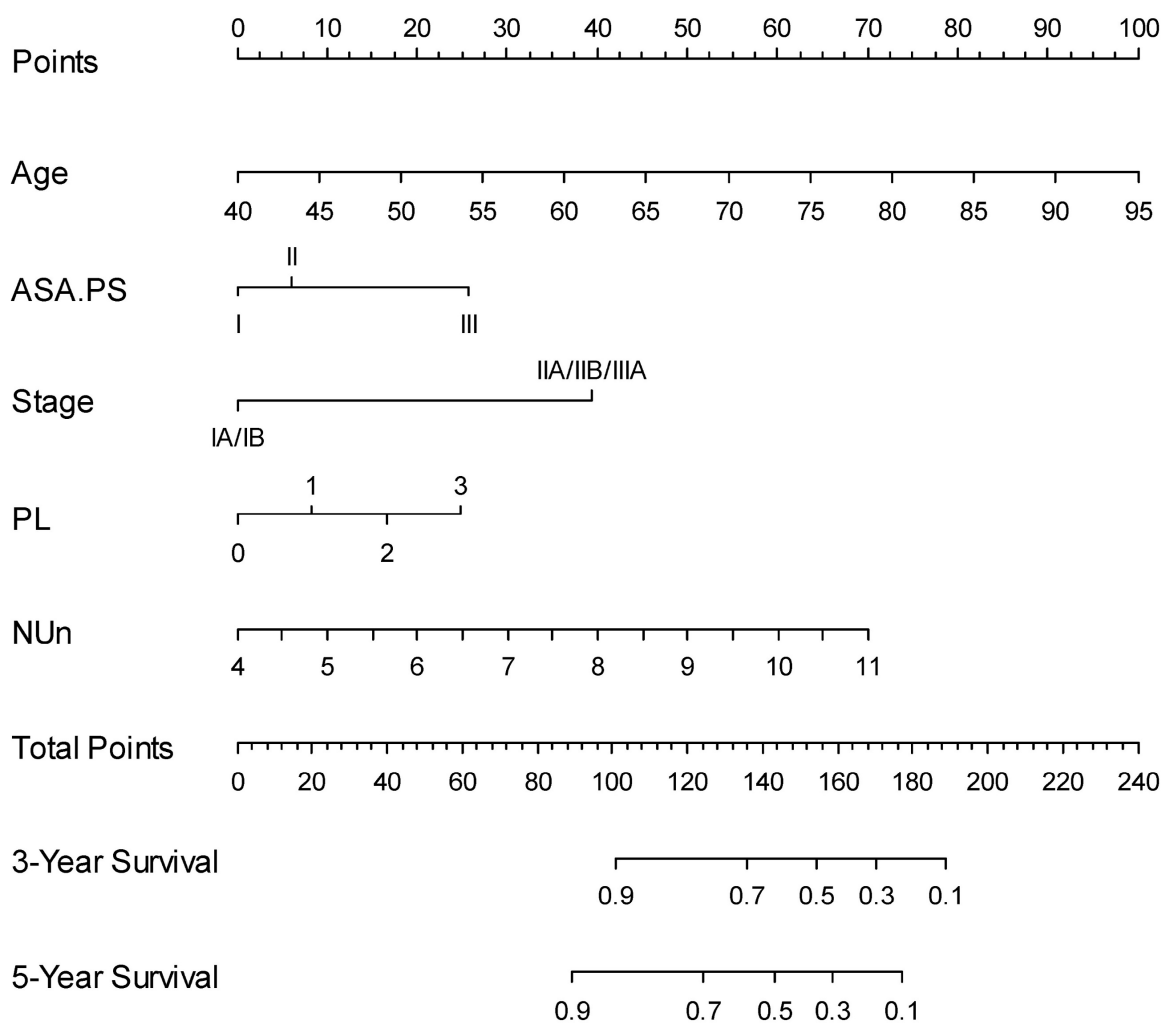
Predictive Nomogram for Overall Survival Based on the NUn Model. ASA.PS: American Society of Anesthesiologists Physical Status; NUn: Noble and Underwood score; PL: pleural invasion.

**Figure 5:**
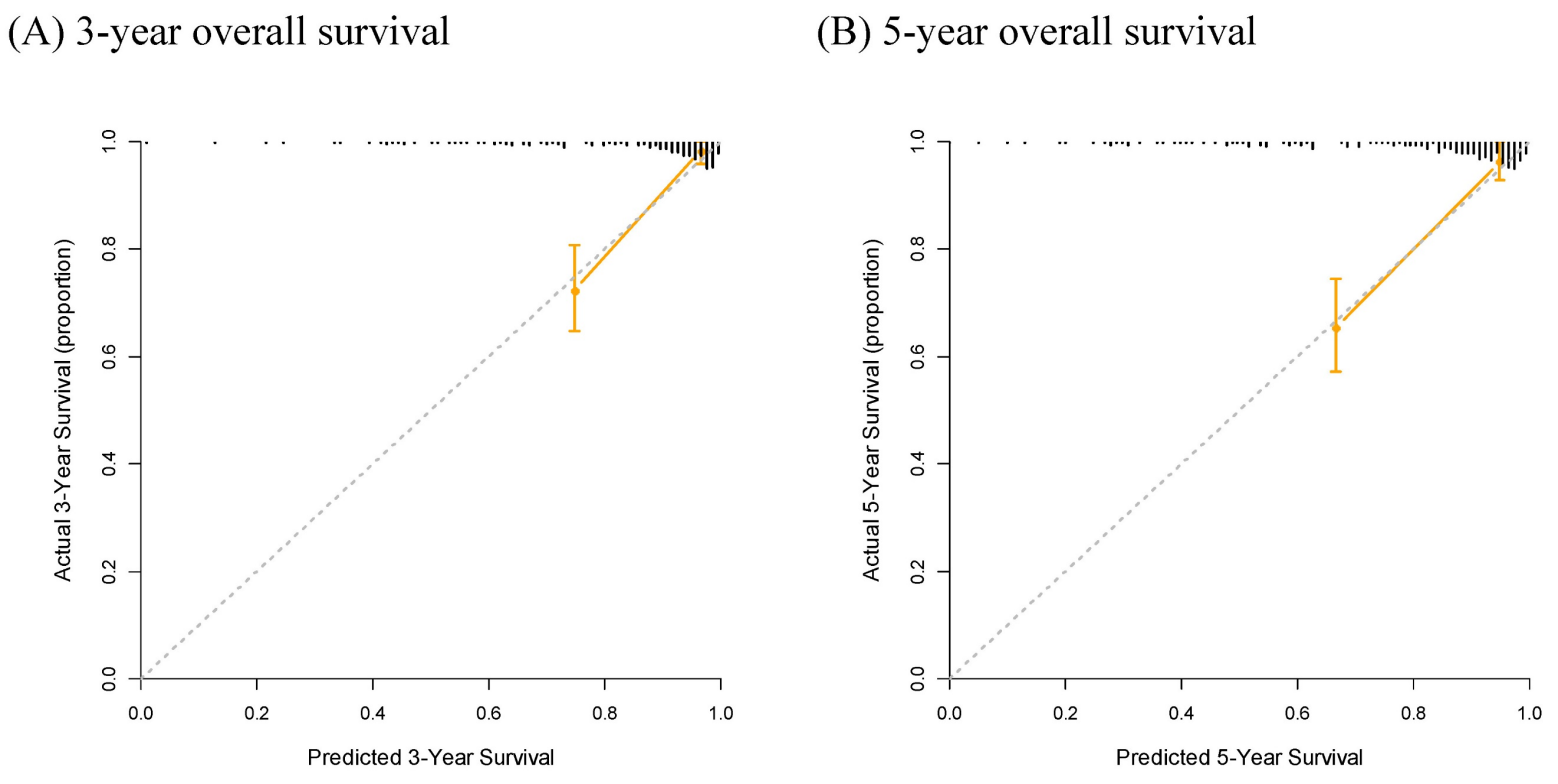
Calibration Curve Analysis for Predicting 3-Year (A) and 5-Year (B) Overall Survival.

Finally, a nomogram based on the NUn model for predicting both the 3- and 5-year survival outcomes was developed (Figure 4). Calibration curves showed that the predicted survival closely aligned with the actual survival probabilities (Figure 5).

## Discussion

Our findings highlight the prognostic value of the NUn score in predicting OS among patients with stage I to IIIA NSCLC. The NUn model, which integrates the NUn score with other clinical variables, demonstrated significantly superior discriminative ability compared with the TNM stage alone across multiple metrics, including C-index, iAUC, AUC, IDI, cNRI, and DCA. Incorporating the NUn model into clinical practice can enhance the prognosis and management of patients with NSCLC, offering a more nuanced and effective approach to treatment planning and follow-up.

The NUn score, which incorporates CRP and ALB levels and WBC counts, effectively captures the key aspects of systemic inflammation and nutritional status, which are both critical determinants of cancer progression and patient outcomes. CRP is one of the most frequently used markers of systemic inflammatory responses in the body 12, 13. In patients with malignant tumors, CRP levels are modulated by cytokines, particularly interleukin-6, which is produced by tumor or surrounding cells 14. The role of CRP in tumorigenesis has been elucidated in various malignant tumors 15. Furthermore, preoperative CRP has been suggested as a significant determinant of survival outcomes of patients with NSCLC 3, 11, 16-18. ALB is indicative of poor nutritional status and systemic inflammation 19-21. Yang *et al.* suggested that ALB is associated with the risk of hepatoma, colorectal cancer, and lung cancer 45. ALB has been suggested as a significant determinant of survival outcomes of patients with NSCLC 3, 22-25. Tumor-related leukocytosis, reflected by an elevated WBC count, is a paraneoplastic syndrome occasionally encountered in the clinical course of patients with lung cancer. Autonomous production of hematopoietic cytokines, such as granulocyte-colony stimulating factor, granulocyte-macrophage-colony-stimulating factor, and interleukin-6, has been identified in some of these patients 46. WBC count has been suggested as a significant determinant of survival outcomes of patients with NSCLC 8, 47, 48. In summary, elevated CRP levels and WBC counts reflect an inflammatory response that can promote tumor growth and metastasis, while low albumin levels indicate malnutrition and chronic inflammation. Integrating these markers into the NUn score provides a holistic view of the patient's physiological state and offers a more accurate prognostic assessment than using a single marker alone.

In line with previous findings, we found strong correlations between the NUn score and various inflammatory and nutritional parameters (ALB, CRP, WBC count, ANC, AMC, and NLR), underscoring the relevance of the NUn score as a comprehensive prognostic marker for survival outcomes. Moreover, the lack of multicollinearity between the NUn score and other variables, such as ASA-PS, TNM stage, and pleural invasion, confirmed its robustness as an independent prognostic factor.

In addition to the NUn score, age, ASA-PS, TNM stage, and pleural invasion were significant predictors of OS in the present study. Age is a well-known prognostic factor, with older patients often having poorer outcomes because of comorbidities and reduced physiological reserve 4-6, 49. The ASA-PS classification assesses preoperative physical health, with higher scores indicating an increased perioperative risk and worse survival outcomes in patients with NSCLC 49. The TNM staging system remains the most critical determinant of prognosis in NSCLC, with higher stages correlating with lower survival rates owing to the challenges in achieving complete surgical resection 3-6, 8. Pleural invasion is a significant predictor of poor prognosis, indicating advanced disease and higher recurrence rates 4, 5.

Integrating the NUn score, age, ASA-PS, TNM stage, and pleural invasion into a unified prognostic model (the NUn model) offers a comprehensive assessment of patient prognosis. The VIFs for these variables (1.03 for age, 1.04 for ASA-PS, 1.11 for TNM stage, 1.09 for pleural invasion, and 1.04 for the NUn score) indicated low multicollinearity, ensuring the robustness of the model. Additionally, these variables did not violate the assumption of proportional hazards. When a nomogram based on the NUn model was developed for predicting both 3- and 5-year survival outcomes, the calibration curves showed that the predicted survival closely aligned with the actual survival probabilities. This nomogram enabled the prediction of individual patient survival outcomes before surgery.

In the multivariate Cox regression analysis, the NUn score emerged as a significant determinant of OS, whereas its individual components (CRP, ALB, and WBC) were not. This indicates that the NUn score had a higher predictive value for OS than CRP, ALB, or WBC count alone in our cohort. Similarly, in the present study, although the CAR, CALLY index, OPS, and mGPS—which heavily rely on CRP and ALB—were significant in the univariate Cox regression analysis, they were not significant in the multivariate Cox regression analysis. These results suggest that a model incorporating the NUn score is more effective for predicting survival than models using individual markers (CRP, ALB, and WBC) or those primarily based on CRP and ALB (such as CAR, CALLY index, OPS, and mGPS), underscoring its promising prognostic value for NSCLC.

Compared with the baseline model based solely on TNM stage, the NUn model demonstrated significantly higher C-index and iAUC values, indicating its improved discriminative ability. The 3- and 5-year OS AUCs were also significantly higher for the NUn model than for the baseline model. Using the IDI and cNRI metrics, the NUn model showed notable improvements in discrimination at both 3 and 5 years, enhancing clinical decision-making. DCA for 3- and 5-year OS indicated that the NUn model provided a higher net benefit than the baseline model, reflecting its better predictive accuracy and clinical utility of the NUn model.

We recognize that the individual components of the NUn score—CRP, ALB, and WBC count—have been previously studied in the context of lung cancer prognosis. However, our study's novelty lies in the creation and validation of the NUn score as a composite, integrated prognostic tool that combines these markers into a single score. By doing so, the NUn score offers a more holistic and accurate assessment of a patient's prognosis. The model incorporating the NUn score showed significantly better performance compared to the use of the TNM staging system alone, suggesting that the NUn score captures critical information about systemic inflammation and nutritional status that is not fully reflected in traditional staging systems. This is an important advancement because it provides clinicians with a more nuanced tool for risk stratification and personalized treatment planning.

The integration of the NUn model into clinical practice can significantly affect risk stratification, treatment planning, monitoring, and prognostication. Preoperatively, the NUn model can identify high-risk patients who may have poorer postoperative outcomes, aiding in informed decisions regarding surgical approaches and the need for preoperative optimization. High-risk patients may require more aggressive adjuvant therapies, such as additional immunotherapy with chemotherapy, to address the higher recurrence risks. Regular postoperative assessment of the NUn model can help track recovery and detect early health changes, thereby facilitating timely interventions. The NUn model provides valuable prognostic information for counseling patients and their families, helping them set realistic expectations and make informed treatment decisions. The NUn model can also serve as a criterion for stratifying patients in clinical trials, thereby ensuring a more personalized approach to patient care. In summary, integrating the NUn model into the clinical management of NSCLC can improve personalized care, optimize treatment outcomes, and enhance the overall quality of care for patients. This comprehensive approach ensures that both the oncological and overall health needs are addressed, ultimately improving the prognosis and quality of life for patients with NSCLC.

The main strength of this study lies in being the first to apply the NUn score to NSCLC, offering new insights into its potential utility. Another strength was the use of a robust dataset comprising a wide range of clinicopathological and laboratory variables, which enhanced the reliability and validity of the findings. This study employed well-established models and statistical methods to ensure robustness and credibility. The use of bootstrap resampling with 1,000 iterations strengthened the reliability and generalizability of the results. An important methodological choice in our study was to use the NUn score as a continuous variable rather than dichotomizing it. This decision was made to preserve the full spectrum of information and maintain statistical power. We believe that this approach maximizes the ability to detect meaningful relationships between the NUn score and clinical outcomes, and helps avoid the potential pitfalls of overfitting, especially given our sample size. Continuous variables, in our view, are less prone to overfitting and more likely to yield findings that are generalizable to broader patient populations. Additionally, we think that continuous measures often provide more actionable insights in clinical practice, allowing for more personalized and precise prognostic assessments based on the full range of NUn score values 42-44. Finally, the development of a nomogram offers a practical tool for predicting OS in patients with NSCLC, facilitating personalized care.

However, this study had certain limitations. As a retrospective study, it was subject to inherent biases that could have affected its generalizability. The single-institution setting may have limited generalizability to other populations. Despite internal validation, the absence of external validation with independent cohorts may have reduced confidence in the model's applicability to other populations. Unmeasured confounding variables could have influenced outcomes and predictive accuracy. We acknowledge the importance of cost-effectiveness in clinical decision-making. The diagnostic costs of the tests used in our study (CRP, ALB, and WBC with differential counts) were relatively low, totaling 12.19 USD for NUn, CALLY, and OPS, and 6.73 USD for CAR and mGPS. These tests are part of standard pre-operative evaluations at our hospital and many teaching hospitals, so they do not incur additional costs. However, we recognize the need for a formal cost-effectiveness analysis to compare these models more rigorously. Future studies will incorporate such analyses to further validate and refine our prognostic models.

In summary, our findings highlight the prognostic value of the NUn score in predicting OS in patients with stage I to IIIA NSCLC. The comprehensive model (NUn model) integrating the NUn score with other clinical variables showed significantly superior discriminative ability compared with TNM stage alone across multiple metrics. Incorporating the NUn model into clinical practice can enhance the prognosis and management of patients with NSCLC, offering a more nuanced approach to treatment planning and follow-up. Future studies should focus on external validation using independent cohorts to confirm the broader applicability and reliability of the model.

## Author contributions

According to the recommendation of ICMJE, Soomin An, Wankyu Eo, Dae Hyun Kim, and Sookyung Lee contributed as authors.

Soomin An, Wankyu Eo, Dae Hyun Kim, and Sookyung Lee contributed to the conception or design of the work.

Soomin An, Wankyu Eo, Dae Hyun Kim, and Sookyung Lee contributed the acquisition, analysis, or interpretation of data for the work.

Soomin An, Wankyu Eo, Dae Hyun Kim, and Sookyung Lee drafted the work or critically revised it for important intellectual content, finally approved the version to be published, and agreed to be accountable for all aspects of the work in ensuring that questions related to the accuracy or integrity of any part of the work are appropriately investigated and resolved.

## Data access statement

The datasets generated and/or analyzed during the current study are available from the corresponding author upon reasonable request.

## Ethical compliance

All procedures performed in this study involving human participants were in accordance with the ethical standards of the institutional and/or national research committee and with the 1964 Helsinki Declaration and its later amendments or comparable ethical standards.

## Figures and Tables

**Table 1:** Inter-group Comparison of the Noble and Underwood Scores Across Different Categorical or Ordinal Variables

Variables	*n* (%)	Median (IQR)	P-value†
**Sex**			
Men	152 (63.1)	5.64 (5.23-6.10)	<0.001
Women	89 (36.9)	5.31 (4.93-5.71)	
**ASA-PS**			
I	22 (9.1)	5.21 (4.65-5.54)	0.007
II	172 (71.4)	5.56 (5.12-6.02)	
III	47 (19.5)	5.58 (5.20-6.41)	
**Smoking history**			
Never	103 (42.7)	5.38 (5.00-5.73)	0.001
Current/former	138 (57.3)	5.65 (5.23-6.12)	
**Histology**			
Squamous	64 (26.6)	6.05 (5.53-6.56)	<0.001
Non-squamous	177 (73.4)	5.42 (5.06-5.73)	
**TNM Stage**			
IA/IB	152 (63.1)	5.50 (5.10-5.80)	0.014
IIA/IIB/IIIA	89 (36.9)	5.60 (5.20-6.40)	
**Type of surgery**			
Segmentectomy	51 (21.2)	5.40 (4.70-5.73)	<0.001
Lobectomy	185 (76.8)	5.55 (5.17-6.07)	
Pneumonectomy	5 (2.0)	6.56 (6.39-9.89)	
**Pleural invasion**			
0	171 (71.0)	5.55 (5.06-6.02)	0.022
1	52 (21.6)	5.39 (5.09-5.74)	
2	6 (2.5)	5.54 (5.08-6.14)	
3	12 (5.0)	6.14 (5.63-6.79)	
**Lymphatic invasion**			
No	206 (85.5)	5.56 (5.13-6.08)	0.174
Yes	35 (14.5)	5.38 (4.99-5.80)	
**Vascular invasion**			
No	225 (93.4)	5.54 (5.09-6.03)	0.982
Yes	16 (6.6)	5.60 (5.17-6.04)	
**Perineural invasion**			
No	235 (97.5)	5.54 (5.09-6.02)	0.273
Yes	6 (2.5)	6.01 (5.45-6.60)	
**Anemia**			
No	159 (66.0)	5.41 (5.05-5.95)	0.001
Yes	82 (34.0)	5.70 (5.32-6.35)	

† Mann-Whiney U tests or Kruskal-Wallis tests ASA-PS: American Society of Anesthesiologists Physical Status; IQR: interquartile range; TNM: tumor-node-metastasis

**Table 2:** The Median Values of Various Continuous Variables and Correlation of the Variables with Noble and Underwood Scores

Variables	Median (IQR)	*r* (P-value)†
Age, years	69.0 (62.0-75.0)	0.276 (<0.001)
BMI, kg/m^2^	23.6 (21.8-25.8)	-0.040 (0.540)
Tumor size, cm	2.8 (2.0-3.7)	0.404 (<0.001)
Albumin, g/dL	4.1 (3.9-4.3)	-0.855 (<0.001)
CRP, mg/dL	0.2 (0.1-0.5)	0.634 (<0.001)
WBC, per μL	6440 (5220-7570)	0.648 (<0.001)
ANC, per μL	3801 (3066-4766)	0.648 (<0.001)
AMC, per μL	492 (390-628)	0.537 (<0.001)
ALC, per μL	1799 (1482-2180)	0.039 (0.545)
Platelet, ×10^3^ per μL	235 (198-281)	0.178 (0.006)
MPV, fL	9.5 (8.8-10.2)	-0.135 (0.036)
NLR	2.1 (1.6-2.9)	0.548 (<0.001)
LMR	3.7 (2.9-4.7)	-0.270 (<0.001)
PLR	132.5 (105.0-163.0)	0.006 (0.926)

† The correlation coefficient of each variable was calculated when comparing it with the Noble and Underwood score. ALC: absolute lymphocyte count; AMC: absolute monocyte count; ANC: absolute neutrophil count; ASA-PS: American Society of Anesthesiologists Physical Status; BMI: body mass index; IQR: interquartile range; LMR: lymphocyte-to-monocyte ratio; MPV: mean platelet volume; NLR: neutrophil-to-lymphocyte ratio; PLR: platelet-to-lymphocyte ratio; TNM: tumor-node-metastasis; WBC: white blood cell

**Table 3:** Univariate and Multivariate Cox Proportional Hazards Regression Analysis for Overall Survival

Variables †	Univariate			Multivariate (NUn model)	
	HR (95% CI)	P-value		HR (95% CI)	P-value
Age, years	1.09 (1.05-1.13)	<0.001		1.07 (1.03-1.10)	<0.001
Sex (female vs. male)	0.41 (0.22-0.76)	0.005			
ASA-PS ‡	2.56 (1.55-4.21)	<0.001		1.87 (1.12-3.11)	0.017
BMI, kg/m^2^	0.99 (0.91-1.07)	0.763			
Smoker (current/former vs. never)	2.10 (1.19-3.70)	0.010			
Squamous (yes vs. no)	3.53 (2.10-5.91)	<0.001			
Tumor size, cm	1.32 (1.18-1.48)	<0.001			
N-stage (1/2 vs. 0)	2.40 (1.36-4.22)	0.003			
TNM stage (IIA/IIB/IIIA vs. IA/IB)	5.58 (3.16-9.84)	<0.001		4.00 (2.19-7.33)	<0.001
Pleural invasion ‡	1.94 (1.49-2.53)	<0.001		1.34 (1.03-1.74)	0.031
Lymphatic invasion (yes vs. no)	2.00 (1.08-3.72)	0.028			
Vascular invasion (yes vs. no)	2.29 (1.03-5.07)	0.041			
Perineural (yes vs. no)	2.34 (0.57-9.64)	0.241			
Albumin, g/dL	0.20 (0.10-0.38)	<0.001			
CRP, mg/dL	1.10 (1.06-1.14)	<0.001			
WBC, per μL	1.00 (1.00-1.00)	0.004			
ANC, per μL	1.00 (1.00-1.00)	<0.001			
AMC, per μL	1.00 (1.00-1.00)	0.002			
ALC, per μL	1.00 (1.00-1.00)	0.140			
Anemia (yes vs. no)	1.29 (0.76-2.18)	0.351			
Platelet, × 10^3^ per μL	1.00 (1.00-1.00)	0.787			
MPV	0.72 (0.58-0.90)	0.003			
NLR	1.31 (1.15-1.49)	<0.001			
LMR*	0.77 (0.64-0.93)	0.007			
PLR	1.00 (1.00-1.00)	0.954			
CAR	1.38 (1.22-1.56)	<0.001			
CALLY index	0.92 (0.87-0.96)	<0.001			
OPS	1.95 (1.46-2.62)	<0.001			
mGPS	2.81 (1.95-4.03)	<0.001			
NUn score	1.78 (1.46-2.16)	<0.001		1.41 (1.15-1.74)	0.001

† The right-hand values in parentheses are the reference values. ‡ Ordinal variables. * Not consistent with the assumption of proportional hazards. ALC: absolute lymphocyte count; AMC: absolute monocyte count; ANC: absolute neutrophil count; ASA-PS: American Society of Anesthesiologists Physical Status; BMI: body mass index; CALLY: CRP‑albumin‑lymphocyte index; CAR: CRP to albumin ratio; CI: confidence interval; HR: hazard ratio; IQR: interquartile range; LMR: lymphocyte-to-monocyte ratio; NLR: neutrophil-to-lymphocyte ratio; NUn score: Noble and Underwood score; OPS: Osaka Prognostic Score; PLR: platelet-to-lymphocyte ratio; TNM: tumor-node-metastasis

**Table 4:** Comparison of the Noble and Underwood Model with Baseline Model for Predicting Survival Outcomes

Metrics	NUn model	Baseline model	Difference	P-value
C-index	0.832 (0.024)	0.720 (0.029)	0.115 (0.022)	<0.001
iAUC	0.802 (0.024)	0.705 (0.019)	0.097 (0.008)	<0.001
AUC 3Y	0.887 (0.028)	0.773 (0.036)	0.114 (0.024)	<0.001
AUC 5Y	0.871 (0.031)	0.740 (0.037)	0.131 (0.028)	<0.001
IDI 3Y	-		0.163 (0.048)	<0.001
IDI 5Y	-		0.179 (0.044)	<0.001
cNRI 3Y	-		0.368 (0.092)	<0.001
cNRI 5Y	-		0.405 (0.085)	<0.001

The values in parentheses are standard errors. The NUn model consists of age, American Society of Anesthesiologists Physical Status, TNM stage, pleural invasion, and NUn score. The baseline model relies solely on the TNM stage. AUC: area under the curve; C-index: concordance index; cNRI: Continuous net reclassification improvement; iAUC: integrated area under the curve; IDI: integrated discrimination improvement; NUn: Noble and Underwood score; TNM: tumor-node-metastasis; Y: year

## Abbreviations

ALB: serum albumin level
ALC: absolute lymphocyte count
AMC: absolute monocyte count
ANC: absolute neutrophil count
AUC: area under the curve
BMI: body mass index
BW: body weight
CI: confidence interval
C-index: concordance index
cNRI: continuous net reclassification index analysis
HR: hazard ratio
IDI: integrated discrimination improvement
IQR: interquartile range
LMR: lymphocyte-to-monocyte ratio
NSCLC: non-small cell lung cancer
NUn: Noble and Underwood score
OS: overall survival
PL: pleural invasion
PLR: platelet-to-lymphocyte ratio
TNM: tumor-node-metastasis
VIF: variance inflation factor

## References

[1] Voorn MJJ, Beukers K, Trepels CMM, Bootsma GP, Bongers BC, Janssen-Heijnen MLG (2022). Associations between pretreatment nutritional assessments and treatment complications in patients with stage I-III non-small cell lung cancer: A systematic review. Clinical nutrition ESPEN.

[2] Balata H, Foden P, Edwards T, Chaturvedi A, Elshafi M, Tempowski A (2018). Predicting survival following surgical resection of lung cancer using clinical and pathological variables: The development and validation of the LNC-PATH score. Lung cancer (Amsterdam, Netherlands).

[3] Liu XY, Zhang X, Zhang Q, Ruan GT, Liu T, Xie HL (2023). The value of CRP-albumin-lymphocyte index (CALLY index) as a prognostic biomarker in patients with non-small cell lung cancer. Supportive care in cancer: official journal of the Multinational Association of Supportive Care in Cancer.

[4] Sato S, Sezaki R, Shinohara H (2024). Significance of preoperative evaluation of modified advanced lung cancer inflammation index for patients with resectable non-small cell lung cancer. Gen Thorac Cardiovasc Surg.

[5] Takada K, Takamori S, Matsubara T, Haratake N, Akamine T, Kinoshita F (2020). Clinical significance of preoperative inflammatory markers in non-small cell lung cancer patients: A multicenter retrospective study. PloS one.

[6] Taylor M, Evison M, Michael S, Obale E, Fritsch NC, Abah U (2024). Pre-Operative Measures of Systemic Inflammation Predict Survival After Surgery for Primary Lung Cancer. Clin Lung Cancer.

[7] Motono N, Mizoguchi T, Ishikawa M, Iwai S, Iijima Y, Uramoto H (2023). Prognostic Impact of Cancer Inflammation Prognostic Index for Non-small Cell Lung Cancer. Lung.

[8] Tomita M, Shimizu T, Hara M, Ayabe T, Onitsuka T (2009). Preoperative leukocytosis, anemia and thrombocytosis are associated with poor survival in non-small cell lung cancer. Anticancer research.

[9] Pellini B, Chaudhuri AA (2022). Circulating Tumor DNA Minimal Residual Disease Detection of Non-Small-Cell Lung Cancer Treated With Curative Intent. J Clin Oncol.

[10] Onaitis MW, Furnary AP, Kosinski AS, Kim S, Boffa D, Tong BC (2018). Prediction of Long-Term Survival After Lung Cancer Surgery for Elderly Patients in The Society of Thoracic Surgeons General Thoracic Surgery Database. The Annals of thoracic surgery.

[11] Hara M, Matsuzaki Y, Shimuzu T, Tomita M, Ayabe T, Enomoto Y (2007). Preoperative serum C-reactive protein level in non-small cell lung cancer. Anticancer research.

[12] Gershov D, Kim S, Brot N, Elkon KB (2000). C-Reactive protein binds to apoptotic cells, protects the cells from assembly of the terminal complement components, and sustains an antiinflammatory innate immune response: implications for systemic autoimmunity. The Journal of experimental medicine.

[13] Yao Z, Zhang Y, Wu H (2019). Regulation of C-reactive protein conformation in inflammation. Inflammation research: official journal of the European Histamine Research Society [et al].

[14] Morris-Stiff G, Gomez D, Prasad KR (2008). C-reactive protein in liver cancer surgery. European journal of surgical oncology: the journal of the European Society of Surgical Oncology and the British Association of Surgical Oncology.

[15] Yang J, Liu Z, Liu H, He J, Yang J, Lin P (2017). C-reactive protein promotes bone destruction in human myeloma through the CD32-p38 MAPK-Twist axis. Science signaling.

[16] Lim JU, Yeo CD, Kang HS, Park CK, Kim JS, Kim JW (2018). Prognostic value of platelet count and lymphocyte to monocyte ratio combination in stage IV non-small cell lung cancer with malignant pleural effusion. PloS one.

[17] Alifano M, Falcoz PE, Seegers V, Roche N, Schussler O, Younes M (2011). Preresection serum C-reactive protein measurement and survival among patients with resectable non-small cell lung cancer. The Journal of thoracic and cardiovascular surgery.

[18] Hara M, Yonei A, Ayabe T, Tomita M, Nakamura K, Onitsuka T (2010). Postoperative serum C-reactive protein levels in non-small cell lung cancer patients. Annals of thoracic and cardiovascular surgery: official journal of the Association of Thoracic and Cardiovascular Surgeons of Asia.

[19] Ishida S, Hashimoto I, Seike T, Abe Y, Nakaya Y, Nakanishi H (2014). Serum albumin levels correlate with inflammation rather than nutrition supply in burns patients: a retrospective study. The journal of medical investigation: JMI.

[20] Crumley AB, Stuart RC, McKernan M, McMillan DC (2010). Is hypoalbuminemia an independent prognostic factor in patients with gastric cancer?. World journal of surgery.

[21] Akula B, Doctor N (2021). A Prospective Review of Preoperative Nutritional Status and Its Influence on the Outcome of Abdominal Surgery. Cureus.

[22] Gupta D, Lis CG (2010). Pretreatment serum albumin as a predictor of cancer survival: a systematic review of the epidemiological literature. Nutrition journal.

[23] Miura K, Hamanaka K, Koizumi T, Kitaguchi Y, Terada Y, Nakamura D (2017). Clinical significance of preoperative serum albumin level for prognosis in surgically resected patients with non-small cell lung cancer: Comparative study of normal lung, emphysema, and pulmonary fibrosis. Lung cancer (Amsterdam, Netherlands).

[24] Tanriverdi O, Avci N, Oktay E, Kalemci S, Pilanci KN, Cokmert S (2015). Pretreatment Serum Albumin Level is an Independent Prognostic Factor in Patients with Stage IIIB Non-Small Cell Lung Cancer: A Study of the Turkish Descriptive Oncological Researches Group. Asian Pac J Cancer Prev.

[25] Ikeda S, Yoshioka H, Ikeo S, Morita M, Sone N, Niwa T (2017). Serum albumin level as a potential marker for deciding chemotherapy or best supportive care in elderly, advanced non-small cell lung cancer patients with poor performance status. BMC cancer.

[26] He D, Yang Y, Yang Y, Tang X, Huang K (2022). Prognostic significance of preoperative C-reactive protein to albumin ratio in non-small cell lung cancer patients: A meta-analysis. Front Surg.

[27] Fujino S, Myoshi N, Saso K, Sasaki M, Ishikawa S, Takahashi Y (2020). The inflammation-nutrition score supports the prognostic prediction of the TNM stage for colorectal cancer patients after curative resection. Surgery today.

[28] Min Y, Li X, Chen H, Xu Y, Lan G (2024). Predicting outcomes of Lung Cancer using the modified glasgow prognostic score: A systematic review and meta-analysis. Pak J Med Sci.

[29] Noble F, Curtis N, Harris S, Kelly JJ, Bailey IS, Byrne JP (2012). Risk assessment using a novel score to predict anastomotic leak and major complications after oesophageal resection. Journal of gastrointestinal surgery: official journal of the Society for Surgery of the Alimentary Tract.

[30] Van Daele E, Vanommeslaeghe H, Decostere F, Beckers Perletti L, Beel E, Van Nieuwenhove Y (2024). Systemic Inflammatory Response and the Noble and Underwood (NUn) Score as Early Predictors of Anastomotic Leakage after Esophageal Reconstructive Surgery. Journal of clinical medicine.

[31] Findlay JM, Tilson RC, Harikrishnan A, Sgromo B, Marshall RE, Maynard ND (2015). Attempted validation of the NUn score and inflammatory markers as predictors of esophageal anastomotic leak and major complications. Diseases of the esophagus: official journal of the International Society for Diseases of the Esophagus.

[32] Bundred J, Hollis AC, Hodson J, Hallissey MT, Whiting JL, Griffiths EA (2020). Validation of the NUn score as a predictor of anastomotic leak and major complications after Esophagectomy. Diseases of the esophagus: official journal of the International Society for Diseases of the Esophagus.

[33] Urabe M, Okumura Y, Okamoto A, Yajima S, Yagi K, Yamashita H (2024). Preoperative NUn score serves as a robust predictor of overall and disease-specific survivals following radical surgery for gastric cancer. Langenbeck's archives of surgery.

[34] Dvorak HF (1986). Tumors: wounds that do not heal. Similarities between tumor stroma generation and wound healing. The New England journal of medicine.

[35] McMillan DC (2009). Systemic inflammation, nutritional status and survival in patients with cancer. Current opinion in clinical nutrition and metabolic care.

[36] Travis WD (2014). The 2015 WHO classification of lung tumors. Der Pathologe.

[37] Detterbeck FC, Boffa DJ, Kim AW, Tanoue LT (2017). The Eighth Edition Lung Cancer Stage Classification. Chest.

[38] Hermanek P, Wittekind C (1994). The pathologist and the residual tumor (R) classification. Pathology, research and practice.

[39] Kawase A, Yoshida J, Miyaoka E, Asamura H, Fujii Y, Nakanishi Y (2013). Visceral pleural invasion classification in non-small-cell lung cancer in the 7th edition of the tumor, node, metastasis classification for lung cancer: validation analysis based on a large-scale nationwide database. Journal of thoracic oncology: official publication of the International Association for the Study of Lung Cancer.

[40] Harrison P, Goodall AH (2016). Studies on Mean Platelet Volume (MPV) - New Editorial Policy. Platelets.

[41] Noris P, Melazzini F, Balduini CL (2016). New roles for mean platelet volume measurement in the clinical practice?. Platelets.

[42] Altman DG, Royston P (2006). The cost of dichotomising continuous variables. BMJ (Clinical research ed).

[43] Naggara O, Raymond J, Guilbert F, Roy D, Weill A, Altman DG (2011). Analysis by categorizing or dichotomizing continuous variables is inadvisable: an example from the natural history of unruptured aneurysms. AJNR Am J Neuroradiol.

[44] Royston P, Altman DG, Sauerbrei W (2006). Dichotomizing continuous predictors in multiple regression: a bad idea. Stat Med.

[45] Yang Z, Zheng Y, Wu Z, Wen Y, Wang G, Chen S (2021). Association between pre-diagnostic serum albumin and cancer risk: Results from a prospective population-based study. Cancer medicine.

[46] Kasuga I, Makino S, Kiyokawa H, Katoh H, Ebihara Y, Ohyashiki K (2001). Tumor-related leukocytosis is linked with poor prognosis in patients with lung carcinoma. Cancer.

[47] Tibaldi C, Vasile E, Bernardini I, Orlandini C, Andreuccetti M, Falcone A (2008). Baseline elevated leukocyte count in peripheral blood is associated with poor survival in patients with advanced non-small cell lung cancer: a prognostic model. J Cancer Res Clin Oncol.

[48] Paesmans M, Sculier JP, Libert P, Bureau G, Dabouis G, Thiriaux J (1995). Prognostic factors for survival in advanced non-small-cell lung cancer: univariate and multivariate analyses including recursive partitioning and amalgamation algorithms in 1,052 patients. The European Lung Cancer Working Party. J Clin Oncol.

[49] Zhang R, Kyriss T, Dippon J, Hansen M, Boedeker E, Friedel G (2018). American Society of Anesthesiologists physical status facilitates risk stratification of elderly patients undergoing thoracoscopic lobectomy. European journal of cardio-thoracic surgery: official journal of the European Association for Cardio-thoracic Surgery.

